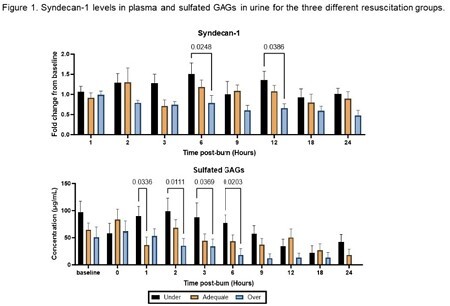# 17 Fluid Resuscitation Effects Endothelial Glycocalyx Shedding in a Swine Model of Severe Burn Injury

**DOI:** 10.1093/jbcr/irae036.017

**Published:** 2024-04-17

**Authors:** Eriks Ziedins, Edward J Kelly, Babita Parajuli, Bonnie C Carney, Jeffrey W Shupp, Lauren T Moffatt, David M Burmeister

**Affiliations:** The Burn Center at Washington MedStar, Washington, District of Columbia; Medstar Washington Hospital Center, Washington, District of Columbia; USUHS, Bethesda, Maryland; MedStar Health Research Institute, Washington, District of Columbia; Georgetown University School of Medicine, Washington, DC; Firefighters’ Burn and Surgical Research Laboratory, Washington, DC; The Burn Center at Washington MedStar, Washington, District of Columbia; Medstar Washington Hospital Center, Washington, District of Columbia; USUHS, Bethesda, Maryland; MedStar Health Research Institute, Washington, District of Columbia; Georgetown University School of Medicine, Washington, DC; Firefighters’ Burn and Surgical Research Laboratory, Washington, DC; The Burn Center at Washington MedStar, Washington, District of Columbia; Medstar Washington Hospital Center, Washington, District of Columbia; USUHS, Bethesda, Maryland; MedStar Health Research Institute, Washington, District of Columbia; Georgetown University School of Medicine, Washington, DC; Firefighters’ Burn and Surgical Research Laboratory, Washington, DC; The Burn Center at Washington MedStar, Washington, District of Columbia; Medstar Washington Hospital Center, Washington, District of Columbia; USUHS, Bethesda, Maryland; MedStar Health Research Institute, Washington, District of Columbia; Georgetown University School of Medicine, Washington, DC; Firefighters’ Burn and Surgical Research Laboratory, Washington, DC; The Burn Center at Washington MedStar, Washington, District of Columbia; Medstar Washington Hospital Center, Washington, District of Columbia; USUHS, Bethesda, Maryland; MedStar Health Research Institute, Washington, District of Columbia; Georgetown University School of Medicine, Washington, DC; Firefighters’ Burn and Surgical Research Laboratory, Washington, DC; The Burn Center at Washington MedStar, Washington, District of Columbia; Medstar Washington Hospital Center, Washington, District of Columbia; USUHS, Bethesda, Maryland; MedStar Health Research Institute, Washington, District of Columbia; Georgetown University School of Medicine, Washington, DC; Firefighters’ Burn and Surgical Research Laboratory, Washington, DC; The Burn Center at Washington MedStar, Washington, District of Columbia; Medstar Washington Hospital Center, Washington, District of Columbia; USUHS, Bethesda, Maryland; MedStar Health Research Institute, Washington, District of Columbia; Georgetown University School of Medicine, Washington, DC; Firefighters’ Burn and Surgical Research Laboratory, Washington, DC

## Abstract

**Introduction:**

Severe burn injury is associated with altered hemodynamics and inflammation that can lead to reduced organ perfusion with subsequent organ dysfunction. While this may be mitigated with aggressive IV fluid administration, both over and under resuscitation can lead to poor outcomes. Confounding matters is endothelial glycocalyx (EGL) damage, which has also shown to be affected by crystalloid administration and is normally represented with syndecan-1 (SDC1) levels. The aim of this study was to examine EGL damage with respect to the amount of fluid administered in a swine model of burn resuscitation.

**Methods:**

Yorkshire pigs were anesthetized and subjected to 40% total body surface area burn and 15% total blood volume-controlled hemorrhage to combat splenic autoresuscitation. Pigs were randomly selected to receive: decision support driven (adequate), fluid withholding (under), and high constant rates (over) of resuscitation. Pigs were monitored for 24 hours post-injury. Blood and urine samples were collected at pre-determined timepoints and analyzed for SDC-1 and sulfated glycosaminoglycans (sGAGs).

**Results:**

A total of 27 pigs were analyzed. Total fluid requirements was significantly elevated in the over group compared to the under resuscitated group (11052 ± 235 mL vs. 794 ± 535 mL, p=0.003), with higher urine output as well (3145 ± 382 mL vs. 861 ± 176 mL, p=0.0008). Lower MAP and higher heart rates at hour 18 post-burn were seen in the under resuscitated group compared to subjects receiving fluids (p=0.003 and p=0.03 for MAP and heart rate, respectively). Kidney damage was also seen with higher blood creatine level at hour 18 post-burn in the under group compared to both the adequate and over resuscitated groups (2.6 ± 0.18, 1.9 ± 0.3, and 1.2 ± 0.15 mg/dL, p=0.02 and p< 0.0001 respectively). There were significantly elevated levels of plasma SDC1 for the under group compared to the over resuscitated group at hour 6 and 12 (p < 0.05 for both) (Figure 1). There were significantly elevated levels of sGAGs for the under group compared to the adequate and over resuscitated groups at hours 1, 2, 3, and 6 post-burn (p < 0.05 for all) (Figure 1).

**Conclusions:**

This animal model showed significant differences in some markers of EGL damage based on varying levels of resuscitation.

**Applicability of Research to Practice:**

Further elucidation of these parameters as they relate to resuscitation may help aid in more individualized care for burn patients.